# Novel spectrophotometric method for the determination of paclitaxel through complex formation with osmium

**DOI:** 10.55730/1300-0527.3763

**Published:** 2025-04-01

**Authors:** Basima A.A. SALEEM, Salim A. MOHAMMED, Amer Th. AL-TAEE

**Affiliations:** Department of Chemistry, College of Science, Mosul University, Mosul, Iraq

**Keywords:** Paclitaxel, osmium, complex formation, spectrophotometry

## Abstract

Paclitaxel (Pac) is an important anticancer bioactive compound, and the availability of an accurate and robust analytical method for its quantification is of utmost importance. In this investigation, a novel, direct, and reliable spectrophotometric method has been proposed for the estimation of Pac via a complex formation reaction with osmium (Os). The method was based on the reaction of Pac with Os tetroxide, which produced a yellowish-brown complex. The resulting complex is soluble in water and displays a maximum absorption peak at 482 nm. The reaction conditions, including reactant concentration, pH, temperature, and reaction time, were optimized to obtain the best spectrophotometric response. A thorough method validation study was conducted, demonstrating that the calibration curve is linear over the concentration range of 1.0–55 μg/mL and follows Beer’s law, with a molar absorptivity of 3.01 × 10^−4^ L/mol^−1^.cm^−1^. The limit of detection (LOD) and limit of quantitation (LOQ) were 0.0098 and 0.0328 μg/mL, respectively. The recovery values were planned and found to be 98.70%–100.23%. At the same time, the precision values (represented by relative standard deviation percent [RSD%]) were better than 0.81% (in injections) and 1.859% (in biological fluids), depending on the Pac concentration. The suggested method was successfully applied to estimate Pac in a pharmaceutical formulation (as injections) and in spiked biological fluids (serum and urine), with results showing good accuracy and precision compared to the reference method.

## 1. Introduction

**Figure 1:**
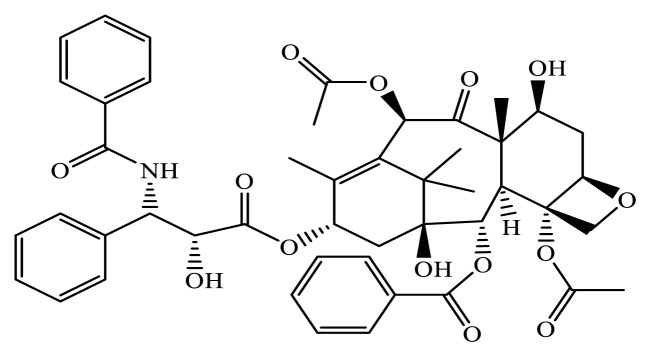
Chemical structure of Pac (C_47_H_51_NO_14_).

Paclitaxel (Pac) is a white to light yellow powder. The chemical formula of Pac is C_47_H_51_NO_14_, with a molecular weight of 853.9 g/mol (Figure 1). It is moderately soluble in water but easily soluble in alcohol [1]. Pac was first discovered and isolated from the bark of the Pacific yew (*Taxus brevifolia*) in 1971 by scientists at the US Environmental Protection Agency. [2]. It was approved for use in the treatment of advanced breast cancer in 1994 by the US Food and Drug Administration [3].

Pac is primarily used to treat various types of cancer, including breast, lung, ovarian, pancreatic, and others [2]. It is given by intravenous injection, usually as part of a multidrug treatment program. Pac inhibits cancer cell division by disrupting microtubules [4]. Accurate quantification of Pac levels is crucial for ensuring effective cancer treatment and monitoring patient response. It is also associated with potential side effects, such as low white blood cell and platelet counts, and nervous system-related complications [5]. Various analytical techniques have been developed and employed for the estimation of Pac, such as biochemical analysis [6], micelle electrophoretic chromatography [7], high-performance liquid chromatography (HPLC) [8–9], liquid chromatography–mass spectrometry (LC/MS) [10], electrophoresis [11], and liquid chromatography–tandem mass spectrometry (LC–MS/MS) [12]. Recent studies have reported a novel method for enhancing sensitivity and selectivity in the determination of Pac, including a three-phase laminar flow microfluidic chip combined with HPLC [13] and high-performance thin-layer chromatography coupled with densitometric analysis (HPTLC-densitometric analysis) [14–15]. Novel electrochemical methods have also emerged as powerful alternatives for the analytical determination of Pac, which have demonstrated excellent sensitivity and selectivity for the detection of Pac, leveraging its electroactive nature through the reversible oxidation–reduction behavior of the Pac core, enabling the quantification of Pac in pharmaceutical formulations and biological matrices using techniques such as differential pulse voltammetry [16] and cyclic voltammetry [17]. Additionally, researchers have explored the development of Pac-loaded polycaprolactone nanoparticles [18], Pac-loaded coated gold nanoparticles [19], and Pac-bonded silver nanoparticles [20] for potential cancer treatment applications. Ultraviolet–visible (UV–Vis) spectrophotometry is one of the simplest techniques used for estimating drug compounds. It is characterized by simple sample preparation, low cost, and analysis that is not time-consuming. Very few UV–Vis spectrophotometric methodologies have been published for the analysis of Pac in bulk. Its pharmaceutical preparations, such as UV spectrophotometry at 230 nm in the presence of phosphate buffer at pH 10 [21] or methanol [22–23], and the first-derivative spectroscopy method have also been used to estimate Pac in the liposomal formulation [24]. This study involves the development of a spectrophotometric method for the determination of Pac in aqueous medium. The method relies on complex formation between Pac and osmium tetroxide (OsO4) as the reagent. The focal aim of this approach is to develop a simple spectrophotometric method for estimating Pac in bulk samples, injections, and biological fluids. The method relies on the formation of a Pac-Os tetroxide complex in aqueous medium.

## 2. Experimental

### 2.1. Instrumentation

All Spectrophotometric measurements and absorption spectra were recorded using a double-beam UV–Vis spectrophotometer (Shimadzu UV-1900i-Japan) equipped with 1.0-cm matched fused silica cells. pH values were measured using a professional Benchtop pH meter (Trans Instruments BP3001-Taiwan).

### 2.2. Chemicals and reagents

High-purity grade chemicals and reagents were used for all experiments.

#### 2.2.1. Pac standard solution (1000 μg/mL)

A total of 0.1000 g of 98% pure Pac obtained from MedKoo Biosciences was weighed and dissolved in 50 mL of ethanol, then diluted to 100 mL with distilled water using a volumetric flask. This solution was stored in a dark container and remained stable for up to one week. This solution was used to prepare a working solution of Pac by simple dilution in a 100-ml volumetric flask [25].

#### 2.2.2. Stock solution of Os (3.93 × 10^−2^ M)

The stock solution was prepared by dissolving 1.00 g of 98% Os tetroxide (Fluka) in 100 mL of distilled water using a volumetric flask. The solution was then transferred to a brown, tightly sealed bottle. The stock Os solution was stored at −5 °C for 48 h prior to use [26].

#### 2.2.3. Working solution of Os (2 × 10^−3^ M)

The working solution was prepared by diluting 5.1 mL of the Os stock solution to 100 mL with distilled water in a volumetric flask. The diluted solution was then transferred to a dark container for storage.

#### 2.2.4. Phosphate Buffer Solution (pH 6.8)

A total of 50 mL of 0.1 M 99% potassium phosphate monobasic solution (KH_2_PO_4_) obtained from BDH was mixed with 22.4 mL of 0.1 M sodium hydroxide solution (Merck). The mixture was then diluted to 100 mL with distilled water using a volumetric flask [27].

#### 2.2.5. Determination of Pac in injections

Vitrax and Pac (Iranian and German) injections are clear, colorless solutions. They are available at a concentration of 100 mg per 16.7 mL. To prepare 100 μg/mL Pac, three injection solutions were mixed well, and 1.67 mL of each mixture was carefully pipetted into a 100 mL calibrated flask. A total of 10 mL of ethanol was added, and the volume was then diluted to the mark with distilled water. Finally, the prepared injection solutions were transferred to an amber vessel for storage and remained stable for 10 days. To estimate the amount of Pac, an aliquot of the diluted injection solution was taken and analyzed according to the standard procedure.

#### 2.2.6. Estimation procedure for the analysis of Pac in biological fluids

The biological fluid samples were prepared as follows [28–29]. For the urine sample, one mL of a freshly drug-free urine sample from a healthy person was spiked with an appropriate amount of Pac standard solution, then diluted 500-fold with distilled water. The final Pac concentrations ranged from one to 55 mg/mL. A serum sample was prepared by taking five mL of drug-free human blood from a healthy volunteer and centrifuging for 30 min at 4000 rpm. To the one mL of the separated filtrate serum, five mL of acetonitrile was added, and the mixture was mixed well to deproteinize it. The sample solution was then centrifuged for five min at 2500 rpm to separate the protein. A convenient amount of Pac standard solution was added and diluted 1000-fold with distilled water to obtain final Pac concentrations ranging from one to 55 μg/mL.

To estimate the Pac in the fluid sample, one to two mL of biological fluids (urine and serum) were collected and analyzed according to the standard procedure.

## 3. Essential procedure and calibration curve

**Figure 2:**
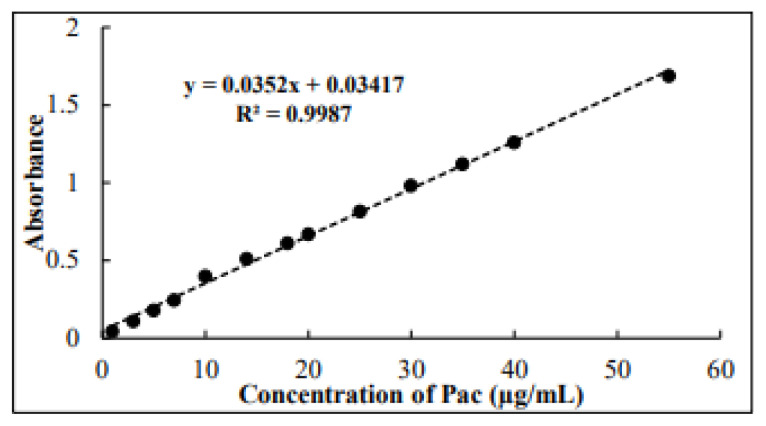
Standard calibration curve for estimating Pac according to the approved method.

Increasing quantities of 0.1–5.5 mL of 100 μg/mL standard Pac solution were added to a series of 10-mL volumetric flasks. To each flask, 2 mL of 2 × 10^−3^ M working Os tetroxide solution and one mL of phosphate buffer (pH 6.8) were added, and the mixture was mixed well. After three min, the flasks were diluted to the marks with distilled water, and the absorbance of each was measured at 482 nm against a reagent blank. The proposed method demonstrated linearity over the Pac concentration range of 1–55 μg/mL, with an excellent coefficient of determination (R^2^ = 0.9987) and a molar absorptivity of 3.01 × 10^−4^ L/mol^−1^.cm^−1^, as shown in Figure 2.

## 4. Results and discussion

### 4.1. Effect of pH

**Figure 3:**
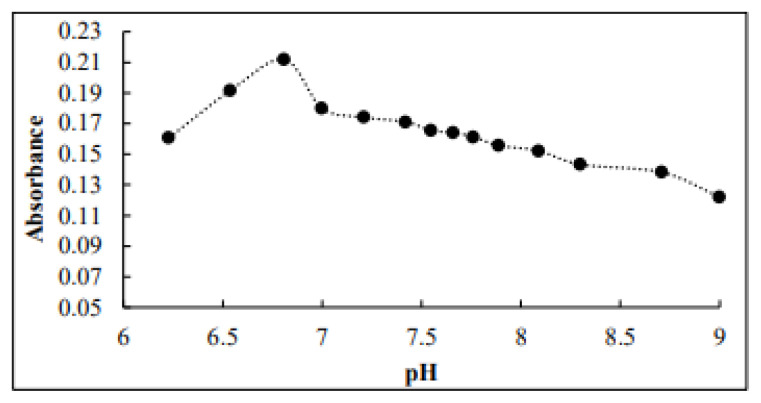
Effect of pH on absorbance of Os-Pac complex.

The effect of pH on the absorbance of the colored product was investigated using a pH meter. The pH measurements indicate that the complex Os-Pac formed at pH 6.8 and exhibited a high absorbance (0.2119) at 482 nm. The results are illustrated in Figure 3.

**Figure 4:**
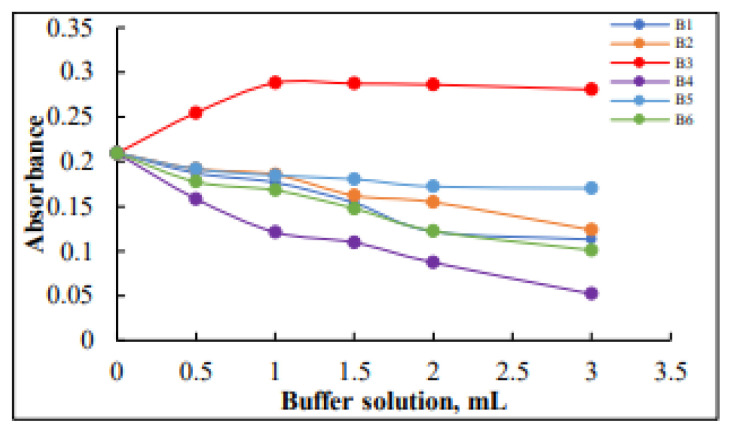
Effect of the amounts of various types of buffer solutions on absorbance.

Different portions (1–4 mL) of various buffer solutions (B_1_ = imidazol-HCl, B_2_ = maleic acid-Tris-NaOH, B_3_ = KH_2_PO_4_-NaOH, B_4_ = triethanolamine-hydrochloric acid-NaOH, B_5_ = sodium hydrogen maleate-NaOH, and B_6_ = Na_2_HPO_4_-NaH_2_PO_4_) of pH 6.8 were studied in terms of their effectiveness on the absorbance of the complex (Os-Pac) formed at a wavelength of 482 nm (Figure 4).

The results in Figure 4 indicate that 1 mL of phosphate buffer solution (B3) at pH 6.8 is the optimal amount to produce the (Os-Pac) complex. Therefore, this volume of the buffer solution was employed in subsequent experiments.

### 4.2. Influence of Os concentration

The influence of one mL of diverse concentrations from 1 × 10^−3^ to 3 × 10^−3^ M of Os tetroxide solutions on the absorbance of the colored product in the presence of 1 mL phosphate buffer solution was studied. The solutions were diluted to the marks with distilled water, and the absorbance of each was measured at 482 nm against its reagent blank. The results are summarized in Table 1 and indicate that a concentration of 2 × 10^−3^ M Os tetroxide is optimal, therefore it was used for the subsequent investigations.

### 4.3. Os amount effect

The effect of adding varying amounts (1–2.5 mL) of Os tetroxide solution (2 × 10^−3^ M) to increasing amounts (50–200 μg) of Pac on absorbance was reviewed. The results, shown in Table 2, revealed that 2 mL of 2 × 10^−3^ M Os tetroxide solution (R^2^ = 0.9929) is the optimal amount for the formation of the Os-Pac complex; therefore, it was used in the recommended procedure.

The influence of various types of surfactant solutions, cetyltrimethylammonium bromide (CTAB), sodium dodecyl sulfate (SDS), cetylpyridinium chloride (CPC) (1 × 10^−3^M), and Triton X-100 (1%), on the absorbance of the complex (Os-Pac) formed was also carried out. The experimental investigations showed that the presence of these surfactant agents adversely affected the absorbance of the resulting complex; therefore, they were excluded from the essential procedure.

### 4.4. Temperature and reaction time effect

**Figure 5:**
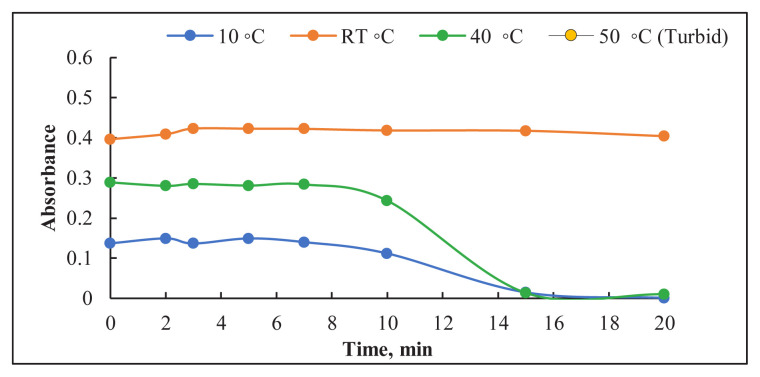
Effect of temperature and reaction time on the absorbance.

The influence of four temperatures (10, 24, 40, and 50 °C) at different time intervals (1–10 min) on the absorbance of the resulting Os-Pac complex was examined using a thermostatic water bath, with temperature monitored with digital thermometers. The results, as shown in Figure 5, confirmed that the reaction of Os with Pac at pH 6.8 required at least 3 min to complete at room temperature (24 ± 2 °C).

### 4.5. Addition order effect

**Figure 6:**
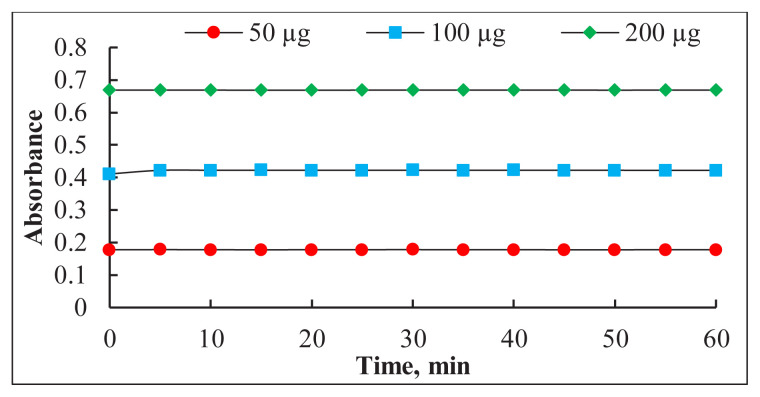
Time influence on the color stability of Os-Pac complex.

The effect of the order of addition of the reaction components on the method’s sensitivity was investigated. The experimental results have proved that the sequence of addition of the reactants denoted as (Pac + Os + buffer solution) to obtain the highest absorbance value (0.4233) of the complex. Under ideal conditions, the effect of time on the color stability of the (Os-Pac) complex was also assessed by measuring the absorbance of three diverse Pac concentrations (5, 10, and 20 μg/mL) at different time points against a reagent blank at 482 nm. As illustrated in Figure 6, the resulting complex remains stable for more than 60 min.

## 5. Final absorption spectrum

**Figure 7:**
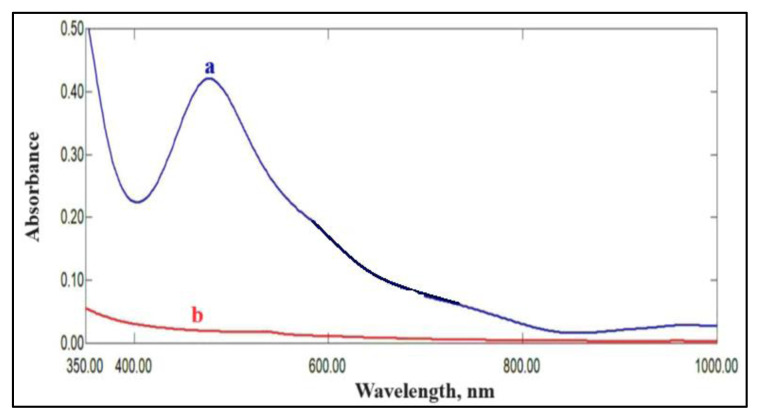
(a) Final absorption spectrum of 4 μg/mL Pac measured vs. blank solution, (b) absorption spectrum of the blank solution vs. distilled water.

After establishing the optimal conditions, as outlined in Table 3, for the reaction of Pac with Os tetroxide in phosphate buffer, the final absorption spectrum of the colored product was plotted in Figure 7.

The spectrum in Figure 7 reveals that the complex Os-Pac formed exhibits a maximum absorption peak at a wavelength of 482 nm, in contrast to its reagent blank solution [a solution containing all components of the reaction except the analyte of interest (Pac)], which shows a low absorbance value at the same wavelength.

## 6. Quantification

Analysis of the Os-Pac complex revealed key metrics, including Beer′s law range, absolute molar absorptivity, recovery percentage, RSD%, limit of detection (LOD), and limit of quantitation (LOQ) values, which were measured and the results are listed in Table 4, indicating that the proposed procedure is both precise and highly sensitive. The linearity of the calibration curve is represented by the regression equation (y = 0.00352x + 0.03417), with a correlation coefficient of 0.9987, highlighting a strong linear relationship between absorbance and concentration. Consequently, the Pac content in pharmaceutical preparations and biological fluids can be accurately determined using this straight-line equation.

## 7. Stoichiometry of the product

Under the ideal conditions of the suggested procedure, the Job and slope-ratio methods have been applied [29] to determine the molar ratio of the resulting Os-Pac complex formed from the reaction of Os with Pac. In both methods, an equal concentration of 1.171 × 10^−4^ M of Pac and Os was used.

**Figure 8:**
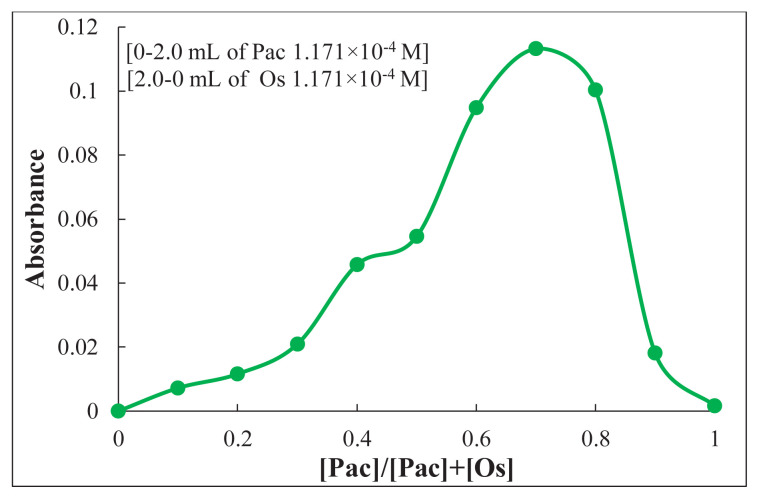
Plot of Job’s method for (Os-Pac) complex formation.

In Job’s method, increasing amounts (0–2 mL) of Pac solution were reacted with complementary quantities (two–0 mL) of Os solution according to the suggested procedure, giving a total volume of two mL in 10 mL calibrated flasks. The solutions were diluted with distilled water, and the absorbance of each was then measured at 482 nm against a reagent blank. The results in Figure 8 show that the mole ratio of the complexes formed is 2:1 (Pac:Os).

**Figure 9:**
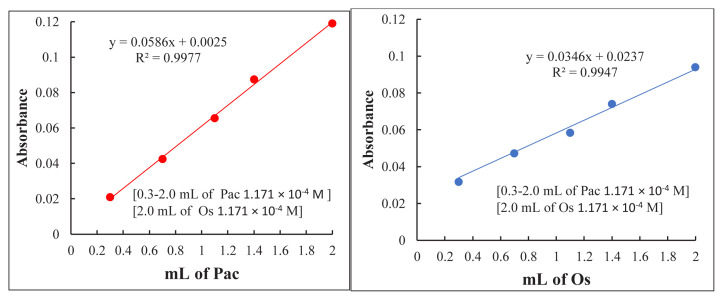
Slope-ratio method curves for the Os-Pac complex.

In the slope-ratio method, two calibration curves were prepared. The first one was prepared by adding increasing amounts of 0.3–two mL of Os solution to a fixed amount of Pac solution (two mL), and in the second curve, increasing amounts of 0.3–two mL of Pac solution were added to a fixed amount of Os solution (two mL) using 10 mL calibrated flasks. The volumes of all flasks were made up to the marks with distilled water, and their absorbance values were measured at 482 nm. The results, illustrated in Figure 9, showed that the Pac: Os ratio in the complex is 2:1.

According to the stoichiometry results, the suggested chemical structure for the (Os-Pac) complex is shown in the Scheme.

## 8. Application

The applicability of the proposed method for the determination of Pac in commercially available fluids (injections) from three different sources, as well as in biological fluids (serum and urine), was studied using three different amounts of 50, 100, and 200 μg of Pac injection (which must be within the range of Beer’s law for the proposed method). The results are listed in Tables 5 and 6.

## 9. Evaluation of the results of the proposed method

The performance of the proposed method for estimating Pac at two concentrations (five and 10 μg/mL) in the pharmaceutical form and biological fluids has been evaluated using a t-test. Table 7 shows that the experimental t-value was better than the tabulated value at the 95% confidence level; the results of the proposed method are not statistically significant. This indicates the feasibility of using the proposed method to determine Pac in drug formulations and biological fluid samples reliably.

## 10. Validity of the proposed method

**Figure 10:**
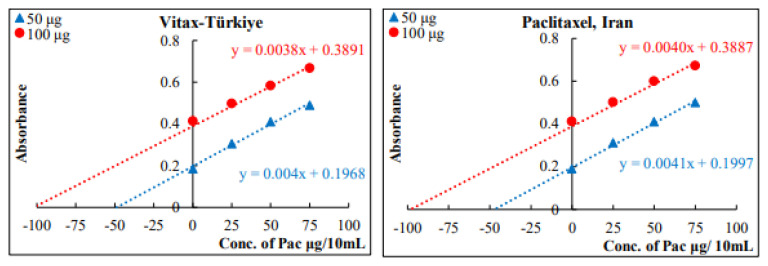
Standard addition plots for the estimation of Pac in the injections.

The standard additions method was applied for the purpose of proving the efficiency and selectivity of the proposed spectrophotometric method for determining Pac in its pharmaceutical preparations (injection forms) and to ensure that it was free from the interference of additives prove that it is free of interferences, The results in Figure 10 and Table 8 indicate that the results of the standard additions method agree well with that results of the proposed method within an adequate range of error.

## 11. Comparison of the proposed methods

Table 9 compares some analytical variables of the recommended method with those of other literature spectrophotometric methods. The suggested method demonstrates high sensitivity and broad applicability.

## 12. Conclusion

A direct spectroscopic method has been proposed to estimate the pharmaceutical compound Pac in pharmaceutical preparations and biological fluids. The method was based on the formation of a water-soluble complex by reacting Pac with Os tetroxide in an aqueous medium at pH 6.8. The resulting complex displayed a maximum absorption peak at 482 nm. The proposed method is sensitive, rapid, and accurate, and does not require complex sample preparation, making it suitable for the routine estimation of Pac. The analytical performance characteristics, such as linearity, accuracy, precision, and selectivity, were evaluated according to the guidelines. Furthermore, the proposed method offers advantages over existing methods in terms of simplicity, cost-effectiveness, and suitability for quality control. Moreover, the proposed approaches do not include temperature control, extraction steps, or specialized equipment. The proposed procedure was effectively employed for the assay of Pac in injections and spiked biological fluids (serum and urine). To assess the study’s originality, this manuscript introduces a novel approach for estimating Pac, highlighting the scarcity of available spectrophotometric methods for its determination. This research addresses a significant gap in the existing literature by offering a visible, reliable, and efficient method for estimating Pac, an area that has received limited attention. The proposed method is characterized by its ease of use, rapid execution, and the stability of the formed complex, all of which are crucial in pharmaceutical analysis, where efficiency and accuracy are paramount. Furthermore, the method demonstrates high selectivity for Pac, even in the presence of interfering substances, making it essential for accurate estimation in biological samples. The ability to accurately quantify Pac in biological matrices is vital for therapeutic monitoring and pharmacokinetic studies, underscoring the relevance of this research for clinical applications. By addressing these critical aspects, this study makes a significant contribution to the field and enhances the methodologies available for Pac estimation.

## Figures and Tables

**Scheme:**
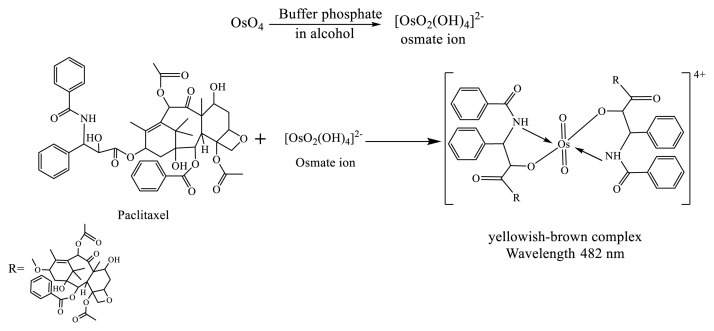
Suggested chemical reaction of Pac with Os.

**Table 1:** Influence of osmium concentration on the absorbance of the resulting complex

Osmium tetroxide (M)	1×10^−3^	1.5×10^−3^	**2×10** **^−3^**	2.5×10^−3^	3×10^−3^
Absorbance	0.2079	0.2881	**0.3621**	0.3544	0.3472

**Table 2:** Effect of osmium amount on absorbance of the complex formed

ml of 2×10^−3^M OsO_4_ solution	Absorbance/μg of Paclitaxel added					R^2^
	50	75	100	150	200	
1.0	0.0732	0.2095	0.2707	0.4318	0.5418	0.9894
1.5	0.1154	0.2432	0.3417	0.4828	0.6099	0.9902
**2.0**	0.1755	0.3123	**0.4021**	0.5534	0.6998	0.9929
2.5	0.1242	0.2858	0.3839	0.5102	0.6684	0.9853
3.0	0.0994	0.2821	0.3775	0.4118	0.6739	0.9583

**Table 3:** Summary of the optimal conditions for the determination of paclitaxel

Parameters	Value
Os concentration (Mole.L^−1^)	2×10^−3^
Os amount (mL)	2
PH	6.8
Type of buffer used	Phosphate
Volume of buffer used (mL)	1
Selected maximum wavelength, λ_max_ (nm)	482
Time needed to form Os-Pac complex	3
Temperature (°C)	RT
Stability of Os-Pac complex period time (minute)	60 <

**Table 4:** Calculated analytical values for the proposed method

Parameters	Value
Linearity range, μg /mL	1 −55
Molar absorptivity, L/mol.cm	3.01×10^4^
Sandell’s sensitivity, μg/cm^2^	0.0284
LOD, μg /mL	0.0098
LOQ, μg /mL	0.0328
Slope (b)*	0.0352
Intercept (a)*	0.03417
Determination coefficient (R^2^)	0.9987

* Regression equation Y = a X + b, where Y is [Pac] in μg/ml.

**Table 5:** Estimation of Pac in the injections by using the proposed method.

Dosage	Certified value	Pac added (μg)	Pac found (μg) *	Reco. *(%) ± RSD*(%)	R.E.* (%)	Measured value (mg)
Vitax (Türkiye)	100 mg/mL 16.7mL	50	49.14	98.28 ± 0.76	−1.72	98.28
		100	100.34	100.34 ± 0.44	0.34	100.34
		200	202.18	101.09 ± 0.27	1.09	101.09
Pac (Iran)	6 mg/mL	50	48.71	97.42 ± 0.81	−2.58	5.85
		100	98.32	98.32 ± 0.46	−1.68	5.90
		200	200.86	100.43 ± 0.56	0.86	6.03
Pac (Germany)	6 mg/mL	50	49.60	99.19 ± 1.31	−0.81	5.95
		100	100.05	100.05 ± 0.43	0.05	6.00
		200	201.34	100.67 ± 0.75	1.34	6.04

* Average of five estimations

**Table 6:** Estimation of Pac in the biological fluids.

sample	mL of sample	Pac found (μg) *	Reco. *(%) ± RSD*(%)	R.E.*(%)
Serum	1	49.34	98.67 ± 0.081	−1.33
		96.98	96.98 ± 0.271	−3.02
		194.9	97.45 ± 0.263	−2.55
	2	51.83	103.66 ± 1.859	3.66
		100.46	100.45 ± 0.090	0.45
		204.92	102.58 ±0.665	2.58

Urine	1	48.44	96.87 ±1.751	−3.13
		97.02	97.02 ±0.092	−2.98
		204.76	102.38 ±0.673	2.38
	2	48.87	97.74 ±0.431	−2.26
		102.12	102.12 ± 1.192	2.12
		202.56	101.28 ± 0.607	1.28

* Average of five estimations

**Table 7:** Evaluation the proposed method in pharmaceutical form and biological fluids.

Sample	Pac added (μg)	Pac found (μg)*	t-exp.#
**Vitax (Türkiye)**	50	50.21	1.039
	100	99.97	0.145
**Pac (Iran)**	50	50.18	0.661
	100	100.15	0.433
**Pac (Germany)**	50	49.82	0.380
	100	99.57	0.758
**Serum** **	50	49.24	1.176
	100	98.87	1.973
**Urine** **	50	49.36	2.066
	100	101.56	1.869

* Average of five estimations,

** 2 mL of sample used.

# $t=(\bar{X}-\mu )\frac{\sqrt{N}}{s}$, tabulated t-value at 95% confidence level is equal to 2.776, with degrees of freedom (N−1 = 4).

**Table 8:** Standard additions method for the estimation of Pac in the injection.

Injection	Certified value	Pac (μg)		Recovery (%)	Measured value (mg)	
	
		Present	Found		Proposed method	Standard addition
Vitax	100 mg/ 16.7mL	50	49.2	98.40	101.41	98.4
		100	102.40	102.40	100.48	102.4
Pac	6 mg/mL	50	48.71	97.42	5.87	5.85
		100	97.23	97.23	5.82	5.83

**Table 9:** Comparison of the proposed method with published methods.

Parameter	Present method	Published method					

		[22]	[23]	[24]	[30]	[31]	[32]
Method	complex formation	solvent system	solvent system	first-derivative		ultraviolet spectroscopic	copolymer nanoparticles
Reagent	Os tetroxide	methanol	methanol	isopropanol	methanol	methanol: phosphate buffer	*PLGA- AFT
l_max_ (nm)	482	230	230	246	230	230	260
Linearity range (μg/mL)	1–55	2–20	1.28 ± 0.346 mg/mL	6–24	5–55	2–20	80–120%
ɛ_max_ (l/mol.cm**)**	3.01 × 10^4^	----	-----	----	---	-----	-----
pH	6.8	7.4	-----	----	10	------	-----
Buffer solution	phosphate buffer	phosphate buffer	------	----	phosphate buffer	7.4	------
Color of the product	yellowish-brown	colorless	colorless	colorless	0.7932	colorless	colorless
LOD (μg/mL)	0.0098	----	0.030 mg/mL	0.9969	0.7932	0.715627	-------
LOQ (μg/mL)	0.0328	----	0.090 mg/mL	3.3229	2.4038	2.168565	-----
Recovery range (injections) (%)	97.42–100.34	99.82–100.48	-----	98.97–102.05	-----	99.42–100.77	100.77
Recovery range (fluids) (%)	96.87 to 103.66	------					
RSD (injections) (%)	≤1.31	≤1.19	<0.05	≤1.31	0.5640	1.386	≤2
RSD (fluids) (%)	≤1.86						
Application of the method	Injection, serum, and urine	Tablet	Pac	Liposomal formulation	Pharmaceutical Formulation	Dosage forms	Pac nanoparticles conjugated with a protein vector

* Pac-poly (lactic-glycolic acid) (PLGA) copolymer nanoparticles-alpha-fetoprotein (AFT)
